# Refractive Correction Treatment in Ectatic Corneal Disorders

**DOI:** 10.1155/2018/8569748

**Published:** 2018-07-25

**Authors:** Vito Romano, Luis Fernandez-Vega Cueto, Roberto Zaldivar

**Affiliations:** ^1^Department of Ophthalmology, St Paul's Eye Unit, Royal Liverpool University Hospital, Liverpool, UK; ^2^Department of Eye and Vision Science, Institute of Ageing and Chronic Disease, University of Liverpool, Liverpool, UK; ^3^Instituto Universitario Fernandez-Vega, Universidad de Oviedo and Fundacion de Investigacion on Oftalmologica, Oviedo, Spain; ^4^Instituto Zaldivar, Mendoza, Argentina

Corneal ectasia is a progressive disorder in which microstructural changes within the cornea cause an alteration of its normal gradient curvature and of its biomechanical behaviour. Over the last few years, different treatments have proved to be safe and effective in halting or slowing the corneal ectasia progression and/or in remodelling of the cornea (such as corneal collagen cross-linking and/or intrastromal corneal ring segments).

In this special issue, the latest research about the surgical and parasurgical treatments of corneal ectasia for therapeutic and refractive perspective is discussed, in terms of halting the ectatic process in keratoconus patients, improving the corneal shape, and minimizing the residual refractive error. An overview is presented of the last 20 years of outcomes and complications for the conservative management of keratoconus (glasses and contact lenses). Cross-linking can halt the disease progression, and intrastromal corneal ring segments can improve the corneal shape and hence the visual quality and reduce the refractive error.

Combined treatment of intrastromal corneal ring segments and corneal cross-linking may be a successful option to halt progressive keratoconus and improve visual acuity. It must be noted that, in particular corneal morphology, such as in central keratoconus with high corneal asphericity and in paracentral keratoconic eyes, a Ferrara-type intrastromal corneal ring segments implantation reported an improvement of postoperative visual acuity and stability over long-term follow-up [1, 2]. Sequential treatment, such as intrastromal corneal ring segments and an extended range of vision intraocular lens implantation, for patients with keratoconus and cataract seems to be an effective option in terms of visual acuity.

*Vito Romano* *Luis Fernandez-Vega Cueto* *Roberto Zaldivar*
